# A Review of Strawberry Photobiology and Fruit Flavonoids in Controlled Environments

**DOI:** 10.3389/fpls.2021.611893

**Published:** 2021-02-01

**Authors:** Rachael Warner, Bo-Sen Wu, Sarah MacPherson, Mark Lefsrud

**Affiliations:** Department of Bioresource Engineering, McGill University, Sainte-Anne-de-Bellevue, QC, Canada

**Keywords:** LED, light spectrum, secondary metabolite, UV, visible light

## Abstract

Rapid technology development in controlled environment (CE) plant production has been applied to a large variety of plants. In recent years, strawberries have become a popular fruit for CE production because of their high economic and nutritional values. With the widespread use of light-emitting diode (LED) technology in the produce industry, growers can manipulate strawberry growth and development by providing specific light spectra. Manipulating light intensity and spectral composition can modify strawberry secondary metabolism and highly impact fruit quality and antioxidant properties. While the impact of visible light on secondary metabolite profiles for other greenhouse crops is well documented, more insight into the impact of different light spectra, from UV radiation to the visible light spectrum, on strawberry plants is required. This will allow growers to maximize yield and rapidly adapt to consumer preferences. In this review, a compilation of studies investigating the effect of light properties on strawberry fruit flavonoids is provided, and a comparative analysis of how light spectra influences strawberry’s photobiology and secondary metabolism is presented. The effects of pre-harvest and post-harvest light treatments with UV radiation and visible light are considered. Future studies and implications for LED lighting configurations in strawberry fruit production for researchers and growers are discussed.

## Introduction

Strawberry (*Fragaria* × *ananassa*) is a valuable crop cultivated worldwide. All strawberry species belong to the genus *Fragaria* and are members of *Rosaceae*, a family that contains many economically significant crops, primarily fruits such as apple (*Malus domestica*), pear (*Pyrus communis*), and peach (*Prunas persica*). The most cultivated strawberry species produced in North America is *F.* × *ananassa*, arising from breeding between two species: *F.* × *virginiana* and *F.* × *chiloensis* (Stewart and Folta, 2010). Strawberry fruits provide a wide range of sensory elicitation and health benefits to the consumer, including high fiber, micronutrient, and ascorbic acid content (Giampieri et al., 2012; Afrin et al., 2016; Battino et al., 2016). Additionally, strawberry fruits are part of a growing trend that highlights plant-derived antioxidants for their proven health benefits (Nile and Park, 2014). These consumer-liking and health-promoting properties have consequently led to strawberry’s strong economic role in the fruit industry. In 2016, the global total production of strawberries was over US$17.7 billion, and this value has substantially increased over the last 10 years (FAO, 2016).

The production of strawberry fruit and their nutritional value are highly impacted by the surrounding environment; therefore, strawberries are often produced in controlled environments (CEs) where lighting and temperature are controlled (Samtani et al., 2019). The use of artificial lighting is a common approach for flower initiation and improved fruit yield (López-Aranda et al., 2011; Hidaka et al., 2014). The critical photoperiod for strawberry flower initiation varies depending upon cultivars and interactions with temperature (Bradford et al., 2010; López-Aranda et al., 2011; Heide et al., 2013). Inhibition of flower initiation at high temperature has been reported for strawberry plants with flowering habits under different photoperiods (Ito and Saito, 1962; Heide, 1977; Serçe and Hancock, 2005). As such, it is suggested that it is inadequate to classify strawberry cultivars solely based on their flowering habits (i.e., short-day, long-day, and day length-insensitive) without considering temperature effects (Durner et al., 1984). Artificial light properties, including wavelength and intensity, also play an important role in strawberry fruit production and quality (Nadalini et al., 2017; Zahedi and Sarikhani, 2017). For instance, sole blue light treatment enhances strawberry (*F.* × *ananassa* cv. Elsanta) fruit production, approximately 25% more than other light sources (Nadalini et al., 2017). End-of-day 735-nm radiation treatment results in a higher strawberry sucrose level (Zahedi and Sarikhani, 2017). These studies have proven that the use of artificial lighting systems allows growers to optimize fruit production and meet consumers’ sensory desires (Nadalini et al., 2017; Zahedi and Sarikhani, 2017).

Plant-derived antioxidants are produced through secondary metabolic pathways, and act as an essential protective barrier against both biotic and abiotic stressors, including light stress (Pocock, 2015; Nadalini et al., 2017). Secondary metabolites such as flavonoids and quinones protect plants from oxidation caused by free radical scavenging (Lü et al., 2010). The extent of secondary metabolite accumulation further influences plant and fruit features, such as specific coloration and antioxidant properties that consumers adore (Akula and Ravishankar, 2011). The high level of total antioxidant capacity contained in strawberry fruit enables the neutralization of free radicals and reduces oxidative stress in the human body (Afrin et al., 2016). The most prevalent secondary metabolites in strawberry fruits are flavonoids, including anthocyanins (Aaby et al., 2012), which are associated with antioxidative and anti-inflammatory properties. Flavonoids predominantly protect plants from UV radiation (Panche et al., 2016), and anthocyanins protect plants from blue and green light (Landi et al., 2020a). In recent years, known antioxidant properties of strawberry fruit have prompted the rise of its global consumption.

Considering the important role that strawberry plants play in the fruit industry, several reviews on strawberry production have been conducted, including flower initiation, development, handling, flavor profile, and health benefits (López-Aranda et al., 2011; Heide et al., 2013; Afrin et al., 2016; Baicu and Popa, 2018; Yan et al., 2018). However, there is limited information available on the impact of light properties on strawberry productivity and secondary metabolite accumulation. Secondary metabolite accumulation in strawberries is impacted by interactive effects between light wavelengths, developmental stages, and lighting strategies (i.e., pre-harvesting and post-harvesting; Erkan et al., 2008; Kadomura-Ishikawa et al., 2013). To this end, this review attempts to compile and compare available research on the impact of light properties within the wavelength ranges of UV radiation (<380 nm) to the visible light spectrum (380–730 nm) on strawberry fruit production, as well as the major group of secondary metabolites, flavonoid compounds. This may lead to improved strawberry fruit production and quality with additional health values in antioxidant activity, while possibly allowing for knowledge transfer to other berry plants grown in CEs.

## Flavonoid Profile and Function in Strawberry Fruits

Plant secondary metabolites have several functions in light signaling and defending against abiotic stresses (Thirumurugan et al., 2018). The most prevalent class of secondary metabolites in strawberry fruits is phenolic compounds, which have at least one phenol unit (aromatic organic ring) in their chemical structures. Phenolic compounds are further divided into different sub-groups, including coumarins, flavonoids, phenolic acids, and tannins. Flavonoids are widely found in foods and beverages of plant origin (i.e., fruits and vegetables; Rozema et al., 2002; Delgado et al., 2019). Flavonoids are easily recognized as flower pigments – they are responsible for the color and aroma of flowers (Dewick, 2001). Flavonoids can be further sub-classified to different subgroups: anthocyanins, flavonols, and flavanols (Aaby et al., 2012; Alvarez, 2014). Over 10,000 flavonoids have been reported, representing the third largest group of naturally occurred secondary metabolites, after terpenoids and alkaloids (Martens et al., 2010). Most flavonoids absorb wavelengths between 315 and 400 nm; therefore, they play an important role in UV radiation screening and as antioxidants for plants (Kotilainen et al., 2009). Sunlight and UV radiation exposure directly impact the extent of flavonoid accumulation in plants (Downey et al., 2006).

### Anthocyanins

Anthocyanins are the most prevalent phenolic compound found in the outer cell layers of various fruits, constituting up to 40% of total phenols in some strawberry cultivars (Aaby et al., 2012). In strawberry, the major anthocyanin is pelargonidin 3-glucoside, with reported anti-inflammatory effects (Da Silva et al., 2007; Amini et al., 2017). Although anthocyanin accumulation is implicated in UV–B protection (280–315 nm), it also occurs under stress conditions involving visible light and far-red radiation (Carvalho and Folta, 2016; Dou et al., 2017). Anthocyanins are the pigments responsible for coloration in flowers and fruits, often serving as visual signal for insect-mediated pollination and seed dispersers (Turturică et al., 2015). Anthocyanin stability largely depends on light, temperature, pH, and the co-pigmentation with other flavonoids (i.e., flavonols; Martens et al., 2010; Turturică et al., 2015). Anthocyanin color is pH-dependent because of its ionic nature; anthocyanin pigments appear red under acidic conditions and blue under alkaline conditions (Khoo et al., 2017). In strawberry plants, anthocyanin accumulates quickly in the late stages of ripening, beginning when fruits turn from white to red and increase more than 10-fold in red, ripe berries (Kadomura-Ishikawa et al., 2013). These phytochemicals largely contribute to antioxidant capacity, impacting the nutritional benefits of the fruit (Aaby et al., 2012; Kadomura-Ishikawa et al., 2013). About 70% of total antioxidant capacity comes from anthocyanins, highlighting its importance among plant secondary metabolites (Wang and Millner, 2009; Giampieri et al., 2012).

### Flavonols

Flavonols are abundantly found in a variety of fruits and vegetables including apples, grapes, and berries, and are reportedly associated with antioxidant potential and reduced risk of vascular disease in humans (Panche et al., 2016). In cultivated strawberry, the major flavonols are quercetin and kaempferol (Labadie et al., 2020). Flavonols are often the main flavonoids at the beginning of the fruit development, but at the ripening stage the flavonoid pathway switches to anthocyanin production (Chassy et al., 2012). Compared to anthocyanins, flavonols contribute more to antioxidant protection against UV-B radiation (Ferreyra et al., 2012; Zoratti et al., 2014); however, they are more sensitive to light properties (Carbone et al., 2009). Studies have reported that flavonol accumulation is highly reduced under shadow treatment in grape (*Vitis vinifera*) skins and is influenced by light levels in grape berry (*Vitis berlandieri* × *V. vinifera*; Pereira et al., 2006; Matus et al., 2009). Apart from functioning as a tissue-protector against UV radiation, flavonols act as flower pigments that attract and defend against insects (Gronquist et al., 2001). Flavonols influence plants’ responses to gravity, but these effects were observed in mutants only (Owens et al., 2008).

### Flavanols

Flavanols, also called flavan-3-ols, are the most common dietary flavonoids. They are used as functional ingredients in food processing to control microbial levels and provide oxidative stability (Aron and Kennedy, 2008). Flavanols consist of monomeric units (i.e., catechins and epicatechin), in addition to oligomeric and polymeric compounds (proanthocyanidins, also called condensed tannins; Al-Dashti et al., 2018). Like anthocyanins and flavonols, flavanol accumulation is developmental stage-dependent (Zhang et al., 2013). For instance, supplemental UV radiation increases flavanol content during development but not in mature grape berries (*V. vinifera* cv. Cabernet Sauvignon). Flavanols help plants protect against harmful pathogens, such as microbes and fungi, as well as insects and herbivorous animals (Aron and Kennedy, 2008). As for flavanols’ dietary effects, they may improve vascular function and nitric oxide availability, as well as modulate metabolism and respiration (Al-Dashti et al., 2018). Being flavanol polymers, it has been reported that proanthocyanidins possess antioxidative and cardio-preventive properties (Monagas et al., 2010).

## Plant Photomorphogenetic Responses and Flavonoid Biosynthesis Under UV Radiation

Biologically active radiation extends from 300 to 800 nm. UV radiation lies in the wavelength range below 380 nm, followed by the visible spectrum between 380 and 720 nm. Outdoors or in an environment lit without supplemental light, approximate 6% of solar radiation is UV radiation, comprising 95% UV-A radiation (315–380 nm) and 5% of UV-B radiation (280–315 nm). UV-C radiation (<280 nm) does not penetrate to Earth’s surface because of the ozone layer. Currently, the major focus of UV radiation-plant investigations is on the UV-B wavelength range, and the number of studies targeting UV-A radiation is relatively small (Verdaguer et al., 2017).

**Figure 1:**
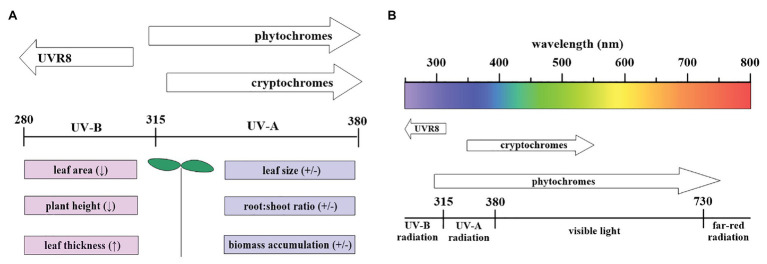
**(A)** Plant responses to UV-A and UV-B radiation, with both positive and negative effects induced by UV-A radiation. **(B)** Plant photoreceptors involved in flower initiation with their corresponding spectral regions, adapted from Folta and Carvalho (2015) and Jones (2018).

Secondary metabolite formation, including phenolic compounds and antioxidants, is a plant’s response to UV-A and UV-B radiation (Caldwell and Britz, 2006), and most flavonoids absorb light in the UV-A radiation range (Cerovic et al., 2002). High levels of UV radiation can cause damage to plants at different levels, including DNA and lipids, leading to impaired gene transcription and photosynthesis (Kunz et al., 2006; Khudyakova et al., 2017). Plant responses to UV-A and UV-B radiation are summarized in Figure 1A. UV radiation alters plant morphology and biomass accumulation during both vegetative and reproductive stages (Müller-Xing et al., 2014; Bernal et al., 2015), and UV-A radiation is perceived by several photoreceptors, including cryptochromes and phytochromes (Figure 1B; Mockler et al., 2003; Folta and Carvalho, 2015). Cryptochromes (cry1 and cry2) are flavin-type blue light photoreceptors (320–500 nm) that have been implicated in numerous developmental and circadian signaling pathways (Banerjee and Batschauer, 2005; Jones, 2018). Phytochromes (phyA to phyE) are light-sensitive proteins with photo-reversible conformers: Pr and Pfr (Folta and Carvalho, 2015). Phytochrome Pr, the inactive form of phytochrome, has a primary absorption peak at 660 nm and a secondary absorption peak located at 380 nm. Absorption peaks of the active form shift approximately 20–70 nm, toward longer wavelengths (408 and 730 nm; Stutte et al., 2009).

The mechanisms underlying this process are poorly investigated in plants, and varied plant responses to UV-A radiation regarding leaf size, morphology and biomass accumulation are reported (Biswas and Jansen, 2012; Kataria and Guruprasad, 2012; Verdaguer et al., 2017). Leaf size is one of the most important determinants of light capture and productivity. Increased rosette size was observed under different accessions of *Arabidopsis thaliana* grown indoors under 1.59 W m^−2^ UV-A radiation and 30 μmol m^−2^ s^−1^ of white light (4,000 K; Biswas and Jansen, 2012). The use of UV-blocking films further revealed that UV-A radiation increases total leaf area in soybean (*Glycine max*) when grown in a greenhouse (Zhang et al., 2014). To the contrary, the solar spectrum, without UV-A and UV-B radiation, induced a larger leaf size when compared to UV-A and UV-B radiation in different varieties of sorghum (*Sorghum bicolor*; Kataria and Guruprasad, 2012). Published data shows no clear link between the impact of UV-A radiation and biomass accumulation, as inconsistent responses have been reported for UV-A-mediated biomass responses (Kataria and Guruprasad, 2012; Zhang et al., 2014). Some studies demonstrated stimulatory effects on biomass accumulation under UV-A radiation (Bernal et al., 2013; Zhang et al., 2014), while others reported inhibitory effects (Kataria et al., 2013). One study concluded that the genotype determines UV-A-mediated responses in plants; however, the study was solely conducted using *A. thaliana* ecotypes (Cooley et al., 2001).

Such contradictory findings may be due to changes in morphology and photosynthetic activity, as well as accumulation of secondary metabolites (Verdaguer et al., 2017). Apart from the impact of plant physiological properties on UV-A radiation, different UV-A radiation conditions might contribute to these conflicting findings. Most UV-A-mediated response studies were conducted with UV-blocking films and the solar spectrum as radiation sources (Zhang et al., 2014; Khudyakova et al., 2017). The use of UV-blocking films only enables the reflection of a certain percentage of UV radiation from solar radiation, and their cut-off wavelengths vary depending on the manufacturer (Katsoulas et al., 2020). In this scenario, although all studies reported the same radiation treatments (i.e., UV-A radiation), radiation spectra might differ. Distinct UV spectra and radiation properties could potentially lead to different plant responses. Furthermore, users cannot select for specific wavelengths passing through the UV-blocking films, as is possible with bandpass optical filters (i.e., blocking UV-A radiation only). As such, UV-A + UV-B treatments are often used as a baseline to compare and discuss the impact of UV-A radiation. Although potential interactive effects between UV-A and UV-B radiation have not yet been reported, they should still be considered and determined with future research.

The most frequently reported UV-B-induced morphological changes are a decrease in leaf area and/or an increase in leaf thickness (Figure 1A; Klem et al., 2012; Robson and Aphalo, 2012; Doupis et al., 2016). UV-B radiation results in leaf changes (i.e., chlorosis, necrosis, and desiccation), and declines in plant height and shoot growth. These observations may serve as a protective mechanism since UV-B can damage photosystems (Gupta et al., 2017). Additionally, secondary metabolite responses differ under UV-B radiation (Schreiner et al., 2012; Hectors et al., 2014). For instance, polyamine and tocopherol levels upregulate quickly (within less than 24 h) in *A. thaliana* (Hectors et al., 2014), whereas flavonoids accumulate at a lower rate, with steady states usually reached after several days (Kusano et al., 2011). Furthermore, dose-dependent responses by flavonoids have been reported; a moderate UV-B dose (ambient radiation) induces flavonoid rutin production, which decreases under both reduced and enhanced UV-B dosage. Ambient UV-B dosage stimulates rutin accumulation, and accumulated rutin is more evident in buckwheat (*Fagopyrum esculentum*) leaves than in flowers (Kreft et al., 2002; Jansen et al., 2008). This suggests that different protectants respond differently based on UV radiation dosage, and that the relative abundance of different flavonoid species differs after UV radiation treatment, implying that distinct dose-response curves for each individual flavonoid compounds exist (Reifenrath and Müller, 2007).

**Figure 2:**
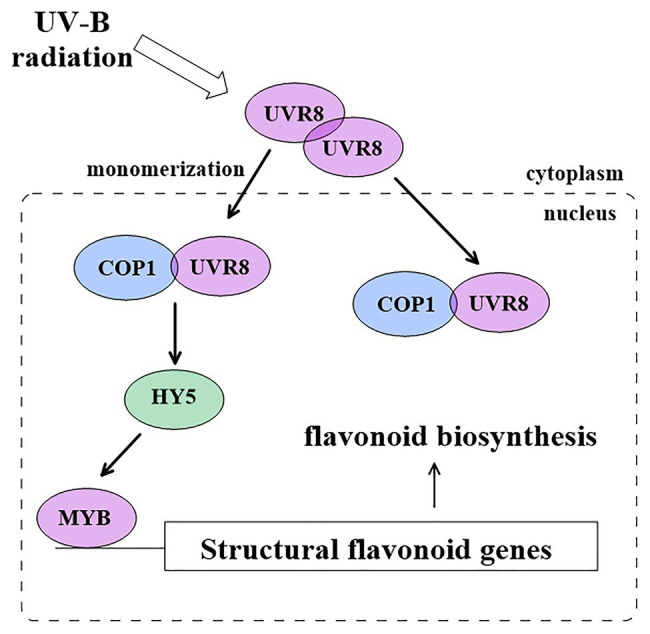
Proposed mechanism for UV-B signaling pathway in flavonoid biosynthesis.

UV-B radiation is perceived by the UV-B photoreceptor UV resistance locus 8 (UVR8), which promotes pest resistance and increases flavonoid accumulation. Under a low dose of UV-B radiation, UVR8 photoreceptor signaling is mediated through the RING-finger type ubiquitin E3 ligase CONSTITUTIVE PHOTOMORPHOGENIC1 (COP1; Figure 2; Lau and Deng, 2012; Peng et al., 2013). COP1 promotes the expression of ELONGATED HYPOCOTYL5 (HY5) in *A.thaliana* (Lau and Deng, 2012) and apple (*Malus* × *domestica*; Peng et al., 2013). Specifically, the presence of low UV-B radiation results in UVR8 monomerization, and UVR8 monomers interact with COP1 to initiate the UV-B signaling pathway. The UVR8-COP1 complex then activates HY5 binding to the promoter region of different R2R3 MYBs, and this leads to flavonoid accumulation in the nucleus (Peng et al., 2013; Jenkins, 2017). Under a high dose of UV-B radiation, UV-B signaling may occur independently of UVR8, possibly *via* mitogen-activated protein kinase (MAPK) signaling (Besteiro et al., 2011).

The negative effects of UV-C radiation on plant development are well established (Urban et al., 2016). Overexposure of UV-C radiation can lead to shortened shelf-life for fresh produce and a reduction in photosynthetic efficiency (De Oliveira et al., 2016; Li et al., 2019). UV-C radiation inflicts considerable damage on lipids and DNA; hence, it is often credited with the most bactericidal activity within the UV wavelength range (Santos et al., 2013). In the context of plant secondary metabolism, it is important to note that UV-C radiation induces the accumulation of phenolics and flavonoids (Nigro et al., 2000; Erkan et al., 2008). However, because of higher energy contained in each photon, the focus on UV-C plant applications for secondary metabolites is largely placed on low doses and on pre‐ and post-harvesting treatments (Urban et al., 2016).

## The Impact of UV Radiation on Strawberry Fruit Flavonoids

Two approaches are often used to manipulate wavelength in CE production: UV-blocking films (i.e., pure polyethylene) and light-emitting diodes (LEDs; Singh et al., 2015; Katsoulas et al., 2020). Both technologies can manipulate wavelengths, yet they have different constraints and effective spectrum ranges. Earlier studies have reported the impact of UV dosage (combined UV-A and UV-B radiation) on strawberry fruit flavonoid levels, predominantly using UV-blocking films (Josuttis et al., 2010; Tsormpatsidis et al., 2011). Strawberry (cvs. Everest, Elsanta) fruits grown under films with high UV transparency (UV-A + UV-B) have higher anthocyanin and phenolic content (cyanidin 3-glucoside, quercetin 3-glucuronide, and kaempferol 3-glucoside) than the strawberry fruits grown under UV-blocking film (Josuttis et al., 2010; Tsormpatsidis et al., 2011). Moreover, UV radiation affects strawberry fruit firmness and color, in which fruit ripened with UV radiation was smaller, firmer, and darker compared to fruit grown under UV-blocking film (Tsormpatsidis et al., 2010; Ordidge et al., 2012). These earlier studies provide insightful information on the impact of UV radiation on strawberry fruit quality and flavonoid contents. However, reported effects on the impact of UV radiation include both UV-A and UV-B radiation. Specific wavelength or radiation treatments within the UV wavelength range cannot be achieved by using solar UV radiation and UV-blocking films. It is unknown if interactive effects within the UV range exist for strawberry flavonoid accumulation.

Unlike UV-blocking films, LEDs offer higher controllability of light properties, such as specific wavelength(s), photoperiod adjustment, and a wide range of intensities (Zoratti et al., 2014; Alrifai et al., 2019; Wu et al., 2019). Many recent studies show the potential of manipulating plant growth and regulating plant secondary metabolite profiles with LED lighting on numerous greenhouse crops within the visible spectrum (Stewart and Folta, 2010; Cocetta et al., 2017; Landi et al., 2020b). In recent years, steady progress has been made regarding wall-plug efficiency and the life-span of UV-LEDs (Kneissl, 2016). UV-LEDs may be superior to UV-blocking films when investigating UV radiation. However, the majority of strawberry studies using UV-LEDs is for enhanced strawberry fruit production, not insect and disease control (Kanto et al., 2009; Suthaparan et al., 2016). To our knowledge, only one study using UV-LED on strawberry (cvs. Maehyang and Seolhyang) flavonoid level has been reported to date, demonstrating that anthocyanin content increased in the Seolhyang cultivar when irradiated with combined 254, 306, and 352-nm LED radiation (Kim et al., 2011). Insufficient data suggest there is a clear lack of studies in this regard, and further study is required to elucidate the impact of narrow UV-A and UV-B spectra on strawberry flavonoids and other secondary metabolite accumulation.

Studies on strawberry secondary metabolites and UV-C radiation have concentrated on pre‐ and post-harvest treatment (Erkan et al., 2008; Xu et al., 2017a, 2019). This might be because of the availability of UV-C radiation sources (i.e., UV discharge lamps with a major peak at 255 nm) and the adverse effect of UV-C radiation on plant development (Kim et al., 2011). Low dose UV-C radiation at the post-harvesting stage has been applied to many valuable crops to reduce postharvest losses due to fungal growth and fruit decay (Marquenie et al., 2002). This UV-C radiation treatment is recognized as an effective method to enhance secondary metabolite concentrations in strawberry fruit within the food industry (Xu et al., 2017a; Saini and Keum, 2018; Li et al., 2019). UV-C radiation improves the antioxidant capacity and reduces softening of fresh-cut strawberry fruit (Erkan et al., 2008; Pombo et al., 2009; Li et al., 2019). Erkan et al. (2008) first observed that low dose UV-C radiation (0.43–4.30 kJ m^−2^) promoted the antioxidant capacity and phenolic content in strawberry fruit storage at 10°C (post-harvest), yet few effects were observed for anthocyanin accumulation. A recent study reported that both total phenolic compounds and anthocyanin in strawberry fruit significantly increased (>20%) with post-harvest treatment using UV-C radiation at 4°C (Li et al., 2019). Low dose UV-C radiation (4.1 kJ m^−2^) slowed down strawberry fruit softening and degradation (Pombo et al., 2009).

Unlike post-harvest treatment, pre-harvest treatment with UV-C radiation is a relatively new approach to improving fruit quality that shows promise (Xie et al., 2015; Severo et al., 2017; Xu et al., 2017a). With UV-C radiation, strawberry fruit exhibit an increase in sucrose, ascorbic acid, and phytochemical profiles (ellagic acid and kaempferol-3-glucuronide). It is possible that UV-C radiation might affect fruit quality *via* the action of plant hormones, as it may be involved in abscisic acid signaling. A recent study showed that pre-harvest UV-C radiation of strawberries had a dose-dependent effect on secondary metabolite levels. Upon harvest, strawberry fruit that underwent UV-C radiation had a higher overall level of anthocyanins and flavonols at 15 kJ m^−2^, and this level dropped to that of the control under the highest UV-C dose (29.4 kJ m^−2^; Xu et al., 2017b). However, pre-harvest UV-C radiation also induced a decrease in volatile compounds responsible for aroma, impacting its flavor profile (Severo et al., 2017; Table 1).

**Table 1:** Strawberry anthocyanin, phenolic, and plant responses under UV radiation compared to control lighting (solar spectrum without UV radiation).

Light spectrum	Cultivar	Intensity and duration	Temperature (°C)	Response	Reference
Solar UV-A + UV-B	Everest and Elsanta	Solar radiation (70% UV transmission)	19.5	Anthocyanin ↑ (cyanidin 3-glucoside) Flavonols ↑ (quercetin 3-glucuronide and kaempferol 3-glucoside)	Josuttis et al., 2010
	Elsanta	Solar radiation (81% UV transmission)	-	Anthocyanin ↑ Flavonoid ↑ phenolic ↑	Tsormpatsidis et al., 2011
	Elsanta	Solar radiation (60–78% UV transmission)	-	Anthocyanins ↑ Phenolics↑ Ellagic acid ↑	Ordidge et al., 2012
UV-C (255 nm)	Candiss	1.70–10.2 kJ m^−2^ (4 min 8 s per week)	-	Early flowering	Forges et al., 2020
	Albion	9.6–15 kJ m^−2^ (18–29 min per 3 days)	20	Anthocyanins ↑ Flavonols ↑	Xu et al., 2017b
	Benihoppe	4.0 kJ m^−2^	4	Total phenolic compounds ↑ Anthocyanin ↑	Li et al., 2019
	Allstar	0.43–4.30 kJ m^−2^ (1, 5, and 10 min, once)	5–10	Antioxidant capacity ↑ Phenolic content ↑ Anthocyanins *Δ*	Erkan et al., 2008
UV LEDs (254, 306, and 352-nm LEDs)	Seolhyang and Maehyang	16.4 W m^−2^ (8 and 16 min per 2 days)	-	Anthocyanins ↑ Phenolics↑ (cv.Seolhyang only)	Kim et al., 2011

All solar UV radiation and UV-light-emitting diode (LED) studies comprise pre-harvest treatment, while all UV-C radiation studies include post-harvest treatment (↑: increase, ↓: decrease).

## The Impact of Visible Light and Far-Red Radiation on Strawberry Development and Flavonoid Accumulation

Key transcription factors for flavonoid biosynthesis have been identified in many fruit crops, such as apple, grapevine, and woodland strawberry (Jaillon et al., 2007; Velasco et al., 2010; Shulaev et al., 2011). These studies indicate that R2R3 MYB transcription factors are the primary regulators of fruit flavonoid biosynthesis in response to changing light conditions within the visible light spectrum. Contrary to its positive regulator role under UV-radiation, COP1 acts as a repressor of flavonoid biosynthesis under visible light (Shulaev et al., 2011; Zoratti et al., 2014). Specifically, COP1 is exported from the nucleus to the cytoplasm, allowing nuclear-localized transcription factors to accumulate and induce expression of genes that are directly regulated by R2R3 MYB transcription factors (Lau and Deng, 2012; Zoratti et al., 2014). Within the visible spectrum, blue light most prominently affects fruit flavonoid accumulation, particularly anthocyanins in unripe strawberries (Zoratti et al., 2014). The relevance of blue light photoreceptors and anthocyanin accumulation was demonstrated at the molecular level, and it was observed that the elevated expression of phototropin 2 FaPHOT2 corresponded to an increase in anthocyanin content (Kadomura-Ishikawa et al., 2013), In addition, blue light leads to overexpression of cryptochrome, resulting in anthocyanin accumulation (Giliberto et al., 2005). Blue light is perceived by cryptochromes that influence different plant developmental steps, including flowering induction and fruit secondary metabolite production (Fantini and Facella, 2020). Increases in flavonoid/anthocyanin biosynthesis with blue light have been reported in tomato (*Solanum lycopersicum* cv. Moneymaker; Lopez et al., 2012) and grape (cv. Malbec; Gonzalez et al., 2015). The impact of blue light and supplemental red light on anthocyanin accumulation has also been investigated in many greenhouse crops (Liu et al., 2018).

Attention on strawberry secondary metabolites is mainly placed on anthocyanin accumulation in CE agriculture, and investigations are mainly conducted with two approaches: (i) sole LED lighting as a growing light with post-harvest treatment; and (ii) different lighting strategies that result in different strawberry phenolic compound/anthocyanin biosynthesis responses (Kadomura-Ishikawa et al., 2013; Miao et al., 2016; Nadalini et al., 2017). Table 2 summarizes current research on the modulation of strawberry flavonoid and fruit productivity under the visible spectrum. Opposed to other greenhouse crops, conflicting data for blue-light-mediated responses and strawberry fruit flavonoid accumulation exist. It appears that blue light is not the most effective wavelength for anthocyanin production in strawberry fruit (Besteiro et al., 2011; Piovene et al., 2015; Nadalini et al., 2017). Blue LED light (e.g., 436 and 470-nm light) induces a higher number of flower clusters and increases final yield for strawberry plants (Nadalini et al., 2017; Magar et al., 2018). A reduction in anthocyanin (pelargonidin-3-glucoside) and phenolic concentrations was observed in strawberry fruit grown under sole 436-nm LED light when compared to the fluorescent light spectrum, with peaks at 430, 450, 540, and 620 nm (Nadalini et al., 2017). Different ratios of 630 and 450 nm (0.7–5.5) LED light has no impact on flavonoid concentrations for strawberry fruit when compared to fluorescent light (Piovene et al., 2015). In the same study, lower flavonoid concentrations were reported in basil leaves (*Ocimum basilicum*) grown under red and blue LED light when compared to basil grown under fluorescent light. The authors concluded that the impact of blue light on flavonoid biosynthesis is species-dependent (Nadalini et al., 2017). Light bandwidth might also attribute to varied responses under blue light. Contrary to other strawberry studies, Miao et al. (2016) reported increased anthocyanins (cyanidin 3-glucoside) in the strawberry cultivar “Yueli” grown under blue plastic film (Miao et al., 2016). Although details of the blue light spectrum used in this study are not known, it is possible that the blue light produced with blue plastic film has a broad spectrum that is similar to the one from UV-blocking films. A broader spectrum of blue light (>25 nm LED bandwidth) could lead to overexpression of both phototropin and cryptochrome, two main blue light photoreceptors. Further investigation into the impact of blue light bandwidths, as well as how blue light influences flavonoid accumulation in different strawberry plant tissues, may answer this question.

**Table 2:** Strawberry anthocyanin, phenolic, and plant responses under the assigned light exposition compared to control (white light produced by fluorescent lamps; ^1^: post-harvesting treatment, ^2^: supplemental lighting, ↑: increase, Δ: same as control, ↓: decrease).

Light spectrum	Cultivar	Wavelength (nm)	Intensity	Temperature (°C)	Response	Reference
Blue light (380–500 nm)	Yueli	blue plastic film	-	-	Anthocyanins ↑	Miao et al., 2016
	Elsanta	436	100 μmol·m^−2^·s^−1^	22–25	Fruit yield ↑ Anthocyanin↓	Nadalini et al., 2017
	Pechka	470^1^	80 μmol·m^−2^·s^−1^	>16	Fruit yield ↑	Magar et al., 2018
Red light (630–700 nm)	Daewang	634 + 661	200 μmol·m^−2^·s^−1^	10–25	Total phenolic compounds ↑ Flavonoids and anthocyanins Δ	Choi et al., 2015
	Yueli	red plastic film	-	-	Anthocyanins ↑	Miao et al., 2016
	Pechka	640^1^	80 μmol·m^−2^·s^−1^	>16	Flower ↓	Magar et al., 2018
	Elsanta	666	100 μmol·m^−2^·s^−1^	22–25	Total phenolic compounds ↓	Nadalini et al., 2017
Purple light (blue + red light)	Albion	449 + 661 (ratio 1:19)	120 μmol·m^−2^·s^−1^	16–21	Fruit yield ↑	Naznin et al., 2016
	Akihime	450 + 660 (ratio 3:7)	60 μmol·m^−2^·s^−1^	25	Fruit yield ↑	Nhut et al., 2003
Far-red radiation	Paros	735^2^	15 μmol·m^−2^·s^−1^	-	Flower ↑ (low temperature)	Zahedi and Sarikhani, 2016
Full spectrum	Fortuna	450 + 650^*^	70–120 μmol·m^−2^·s^−1^	15	Flower ↑	Díaz-Galián et al., 2020
	Fukuoka S6	White LED^2^	>400 μmol·m^−2^·s^−1^	25	Fruit yield ↑	Hidaka et al., 2013

* With narrow-spectrum and broad-spectrum of 650 nm LED light.

In the presence of red light and far-red radiation, activated photoreceptors (phytochromes) repress COP1 function and allow its export from the nucleus, thus inducing flavonoid gene expression (Lau and Deng, 2012; Tossi et al., 2019). The impact of red light on strawberry secondary metabolites and development was reported by Choi et al. (2015), who compared the flavonoid and phenolic compounds of strawberry (cv. Daewang) when grown under 448, 634, and 661-nm LED light in a growth chamber, and with supplementary light in a plastic greenhouse. Upon fruit maturation and testing, higher levels of total phenolic compounds were observed under red light (634 + 661 nm) treatment in the growth chamber, yet there was no significant difference in the amount of anthocyanin and flavonoid between any of the treatments. This was confirmed in a later study with 666-nm LED and strawberry (cv. Elsanta; Nadalini et al., 2017). Similar to the conflicting data obtained with the blue film, opposite anthocyanin responses to “red light” produced with red film and red LED light were reported (Miao et al., 2016). Strawberry (cv. Yueli) grown under red film had a significant impact on total anthocyanin concentration and individual anthocyanins (pelargonidin 3-glucoside and pelargonidin 3-malonyglucoside; Miao et al., 2016). Although the detailed light spectrum under these plastic films was not presented (Miao et al., 2016), differences in secondary metabolite responses to light spectrum imply that specific wavelengths can differentially affect secondary metabolite accumulation in strawberry plants.

Unlike LED light in the visible spectrum, sole far-red radiation has less impact on secondary metabolite accumulation. Yet, it plays an import role in flowering and has proven an effective method for improved flowering with short duration at the end of the day (Zahedi and Sarikhani, 2016, 2017). The authors tested the end-of-day approach with 735-nm LED radiation using different exposure durations and temperatures on various developmental stages for strawberry (cv. Paros). They concluded that flowering can be induced in 12-week old and older strawberry plants through 32 daily cycles of 735-nm LED radiation at a cooler temperature (Zahedi and Sarikhani, 2016). A similar conclusion on flowering initiation was made when end-of-day lighting was combined with mixed red light and far-red radiation (Rantanen et al., 2014).

Although beyond the scope of this review, it is noteworthy to mention that purple light, with different ratios of red and blue LED light as well as intensity, results in increased stolon production, higher photosynthetic activity, and fruit productivity in strawberry plant (Nhut et al., 2003; Wu et al., 2009; Piovene et al., 2015; Naznin et al., 2016). An increase in strawberry flowering and fruit yield occurs with full-spectrum and white LED light (Hidaka et al., 2013; Díaz-Galián et al., 2020). In addition to the purple light spectrum, intensity is critical for influencing strawberry growth (Zhou et al., 2005; Choi et al., 2015). This response appears cultivar-dependent (Smeets, 1976, 1980). An increased daily light integral result in higher dry matter accumulation, propagation efficiency, and quality of strawberry runner plants (Miyazawa et al., 2009; Zheng et al., 2019). In summary, the importance of wavelength and its bandwidth should be emphasized and light spectra with different emitting wavelengths and lighting strategies clearly affect flavonoid content and fruit productivity in strawberry plants. The shift from broad-spectrum to narrow spectrum artificial lighting system is gaining momentum, but there is a paucity of studies comparing broad-spectrum to narrow-spectrum light. This is compounded by conflicting findings for the blue-light-mediated response in strawberry fruit. More CE research could further elucidate the impact of different wavelengths and underlying mechanisms, while determining interactions with temperature-dependent processes, to improve fruit properties, including yield, quality, and nutritional value.

## UV Radiation and Photobiological Safety

UV radiation triggers the accumulation of flavonoids and other secondary metabolites (Caldwell and Britz, 2006). However, widespread use remains elusive, mainly caused by a limited selection of UV radiation sources and the photobiological hazard it presents to humans (Voke, 1999). Several types of UV radiation sources (i.e., gas-discharge lamps, fluorescent bulbs, and LEDs) are not often used in plant photobiology studies. UV gas-discharge lamps radiate a sharp 255-nm spectrum, and are accompanied by many disadvantages, including low radiation output and limited effective radiation area (Sarigiannis et al., 2012). These disadvantages limit further investigation into the impact of UV radiation on plants. Unlike conventional UV radiation sources, UV-LEDs available on the market have a wide range of wavelength selection. By adding aluminum nitride (AlN) to the GaN diodes, emitting UV wavelengths ranges cover from 220 to 380 nm. Unlike earlier UV devices with less than a 100-h lifespan (L50, 50% of light bulbs fail at 100 h), current UV-LEDs emitting at 280–310 nm now boast a lifespan of at least 3,000 h (Würtele et al., 2011; Fujioka et al., 2014; Kneissl, 2016), with some exceeding more than 10,000 h (Glaab et al., 2015). Although these UV-LEDs still have lower reliability and longevity than LEDs in visible spectrum, they have become an emerging radiation source for research involving UV germicidal irradiation, such as water treatment and microbial inactivation (Song et al., 2016; Kebbi et al., 2020).

A major concern when applying UV radiation in plant production facilities is UV photobiology safety. While employing any UV radiation source, there is a potential risk of being exposed to hazardous ocular and skin UV radiation (Ichihashi et al., 2003; Laube et al., 2004), and UV photobiology safety precautions for users are necessary (Lau, 2013). Controls to prevent skin and eye injuries should be placed, and protective housing that restricts or reduces UV exposure is an effective approach. If personnel requires to entering UV-radiation environment, personal protective equipment is highly recommended. Many international bodies have published guidelines that assess and evaluate photobiological eye safety based on wavelength and exposure (European Union, 2006; International Electrotechnical Commission, 2006). For radiation between 180 and 400 nm, the exposure limit is 30 J cm^−2^ within 8-h per day. Note that when users assess the UV radiation environment, spectral weighting functions need to be applied. Sunglasses provide adequate, as most sunglasses are able to greatly reduce the amount of UV radiation (Tuchinda et al., 2006; Wu and Lefsrud, 2018).

## Concluding Remarks and Future Outlook

Here, we review aspects of photobiology and flavonoid accumulation that are relevant to strawberry plant production. Studies showing our interest in enhancing strawberry plant growth, development, metabolites, and crop status span nearly 100 years. Information on photobiology research can be utilized to tailor artificial light spectra, which can target the development of flavonoid content in strawberry fruits. More specifically that different wavelengths will elicit varied responses in the growth and quality of fruit production. Practically applied, optimized light recipes reduce the necessary electrical inputs, while increasing the crop yields and quality. Pre-harvesting UV treatment combined the UV-A to UV-C wavelengths is a powerful tool for stimulating flavonoid biosynthesis in strawberry fruits; however, UV-C radiation alone impacts flavor profile. In post-harvesting treatments, UV-C radiation shows promising results on enhancing secondary metabolites. We expect that UV LEDs will be increasingly used to stimulate desirable fruit metabolites, while requiring only short doses (minutes per day) to elicit a response. Evidence indicates that blue LED light can enhance flavonoid accumulation in greenhouse crops, but this is not explicitly seen with strawberry plants. Blue-light-mediated responses in strawberry fruit flavonoid accumulation are bandwidth-dependent, rather than wavelength-dependent. Based on literature reported, a spectrum with board blue light spectrum or with multiple peaks in the blue wavelength range targeting both phototropin and cryptochrome is optimal and recommended for enhancing flavonoid accumulation. Understanding the action spectrum (or spectral dose-response curve) of flavonoid biosynthesis in different tissues is important to improve the precision of flavonoid production and the antioxidant capacity in strawberry CE production, making these “super foods” more super. Continued investigation of this variation in flavonoid response to light spectrum will provide important knowledge on light signaling machinery, such as COP1-mediated pathways, in strawberry, and possibly other fruit producing species.

Based on research reviewed, we believe there is value in pursuing further research on the implication of light spectra on strawberry secondary metabolites to improve crop quality for human health. The following areas should be considered for further study to fill knowledge gaps: (1) the impact of pre-harvest UV-A on secondary metabolite accumulation. Flavonoids absorb majorly in the UV-A spectrum, yet there is minimal research available on the direct impact of UV-A on flavonoid accumulation in strawberries. (2) Further investigations into the impact of pre-harvest UV-C radiation, considering post-harvest research is very promising. UV-C LED sources with different wavelengths are highly available, and the accessibility to both researchers and producers make results more accessible. (3) Determination of the impact of specific visible light wavelengths across both cultivars and temperature, especially with white LED light. (4) Investigations into blue: red light ratios in purple lighting during vegetative growth and flowering to determine impact on secondary metabolite profile in strawberries. (5) Degradation monitoring of flavonoids during post-harvest storage and transport, to determine timeframe of benefits imposed by augmented light spectrum.

## Author Contributions

RW and B-SW led the writing of this paper. B-SW and SM were the major editors. SM and ML contributed over 40% of the writing for the paper. ML is the correspondence point person. All authors contributed to the article and approved the submitted version.

### Conflict of Interest

The authors declare that this study was funded in part by Gardyn Inc. The funder was not involved in study design, collection, data analysis, the drafting of this manuscript, or the decision to submit it for refereed publication.
